# Using expression arrays for copy number detection: an example from E. coli

**DOI:** 10.1186/1471-2105-8-203

**Published:** 2007-06-14

**Authors:** Dmitriy Skvortsov, Diana Abdueva, Michael E Stitzer, Steven E Finkel, Simon Tavaré

**Affiliations:** 1 Molecular and Computational Biology Program, Department of Biological Sciences, University of Southern California, Los Angeles, CA 90089-2910, USA; 2 Department of Human Genetics, UCLA School of Medicine, University of California, Los Angeles, USA; 3 Department of Pathology, Keck School of Medicine, University of Southern California, Los Angeles, USA; 4 Mount Sinai School of Medicine, New York, NY 10029, USA; 5 Department of Oncology, University of Cambridge, Cambridge, UK

## Abstract

**Background:**

The sequencing of many genomes and tiling arrays consisting of millions of DNA segments spanning entire genomes have made high-resolution copy number analysis possible. Microarray-based comparative genomic hybridization (array CGH) has enabled the high-resolution detection of DNA copy number aberrations. While many of the methods and algorithms developed for the analysis microarrays have focused on expression analysis, the same technology can be used to detect genetic alterations, using for example standard commercial Affymetrix arrays. Due to the nature of the resultant data, standard techniques for processing GeneChip expression experiments are inapplicable.

**Results:**

We have developed a robust and flexible methodology for high-resolution analysis of DNA copy number of whole genomes, using Affymetrix high-density expression oligonucleotide microarrays. Copy number is obtained from fluorescence signals after processing with novel normalization, spatial artifact correction, data transformation and deletion/duplication detection. We applied our approach to identify deleted and amplified regions in *E. coli *mutants obtained after prolonged starvation.

**Conclusion:**

The availability of Affymetrix expression chips for a wide variety of organisms makes the proposed array CGH methodology useful more generally.

## Background

Comparative genomic hybridization (CGH), developed by Kallioniemi et al. [1], has made a significant impact on molecular cytogenetics as a powerful tool for detection of chromosome copy number aberrations. However, CGH to metaphase chromosomes provides limited resolution at the 5–10 Mb level. Solinas-Toldo et al. [2] introduced a matrix-CGH technique in which target DNA was immobilized onto glass slides and hybridized with tumor DNA. Array CGH techniques utilizing printed microarrays or pre-fabricated high density oligonucleotide arrays were further refined to increase the sensitivity to detect single gene gains and losses [3,4]. More recently several genome-wide approaches using high-density oligonucleotide microarrays or SNP chips have been developed [5-10]. Experiments such as these have identified considerable copy number variation across the human genome [11].

Similar chromosomal imbalances are seen in evolving populations of bacteria, where deletions, duplications and amplifications of large regions of the chromosome, some including up to 2% of the genome, are observed [12-14]. In this study, we used the GeneChip *E. coli *Antisense Genome Array to study DNA gene copy number variation in evolving populations of *E. coli*.

When *E. coli *are incubated in batch culture for long periods of time without the addition of nutrients, novel mutants expressing the Growth Advantage in Stationary Phase (or GASP) phenotype appear [15]. These mutants have the ability to outcompete other cells in the population, even though all cells initially had identical genotypes. Previously characterized mutations conferring the GASP phenotype included point mutations, small deletions and insertions, and transposition events [16-18], [S. Finkel, unpublished]. In this study we characterize the larger scale changes associated with deletions and duplications that can include hundreds of kilobases of the genome.

We describe an unconventional approach for copy number detection using commercially available *E. coli *high-density expression arrays that includes raw data normalization, background correction, data transformation based on a physical model of the microarray hybridization process, and deletion/duplication detection using a hidden Markov model approach.

## Results and Discussion

### Affymetrix platform

Although the GeneChip *E. coli *Antisense Genome Array was designed primarily as a tool for expression analysis, it was also created to maximize the amount of *E. coli *K-12 genome sequence covered. In addition to probes for all annotated genes in the *E. coli *genome, probes for small unannotated genes, untranslated RNA, and uncharacterized intergenic regions were included in the design. The array contains probe sets to detect the antisense strand of more than 4,200 known open reading frames. In addition, over 1,350 intergenic sequences were interrogated in both directions, permitting accurate copy number detection of potential genome fragments. *E. coli *Antisense Genome Arrays are an intermediate between classical expression chips and newly developed tiling arrays, which are more suitable for aCGH analysis due to their denser genomic coverage. Array experiments were performed according to a customized protocol; bacterial strains, culture media, and cell growth conditions are described in [19]. The custom sample preparation protocol used in preparing the hybridization cocktail for the Affymetrix experiment was adapted from [20].

### Normalization

**Figure 1:**
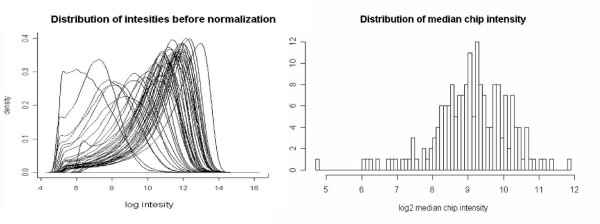
**Pre-Normalized Probe Intensity Distribution**. Summary of data prior to normalization. Distribution of raw signal intensities [left panel], and histogram of median chip intensities [right panel].

Due to variations in DNA yield between preparations of replicate samples, the dataset analyzed here presents an example of systematic errors attributable to variation in biological samples, where median chip intensities vary 100-fold across the dataset. We introduce a new method for normalization, as well as for finding systematic biases associated with variations in experimental conditions; see Figure 1.

**Figure 2:**
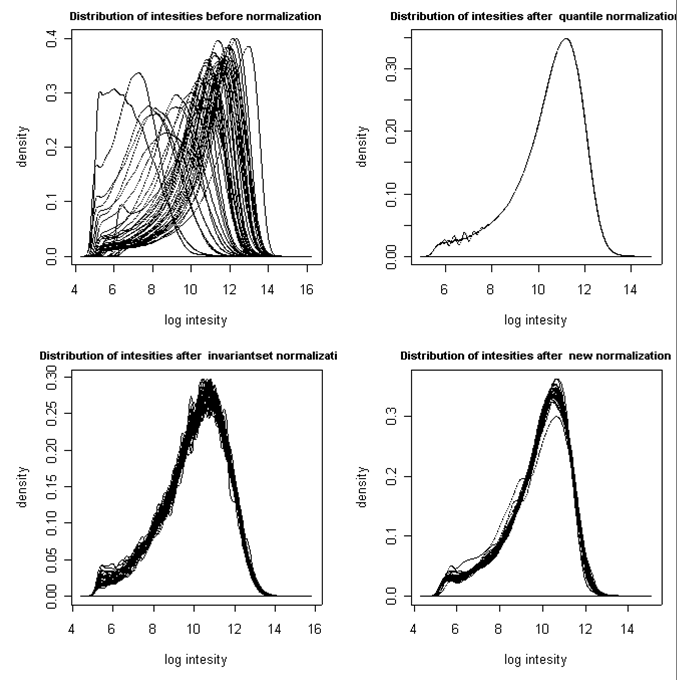
**Comparison of Different Normalization Methods**. Comparison of different normalization methods: distribution of pre-normalized intensities in the dataset [top left], distribution of normalized intensities using quantile normalization [top right], distribution of normalized intensities using invariant set normalization [bottom left], distribution of normalized intensities using proposed normalization routine [bottom right].

The resulting normalized distributions are shown in Figure 2. Visual exploration of the intensity distributions shows that the proposed method (lower right) performs well and does not introduce apparent artifacts as, for example, invariant set normalization (lower left) appears to do. Notice that despite similarity in post-normalized distributions, the signal distributions vary significantly in the tails, where duplications and amplifications are presumably located.

### Spatial normalization

**Figure 3:**
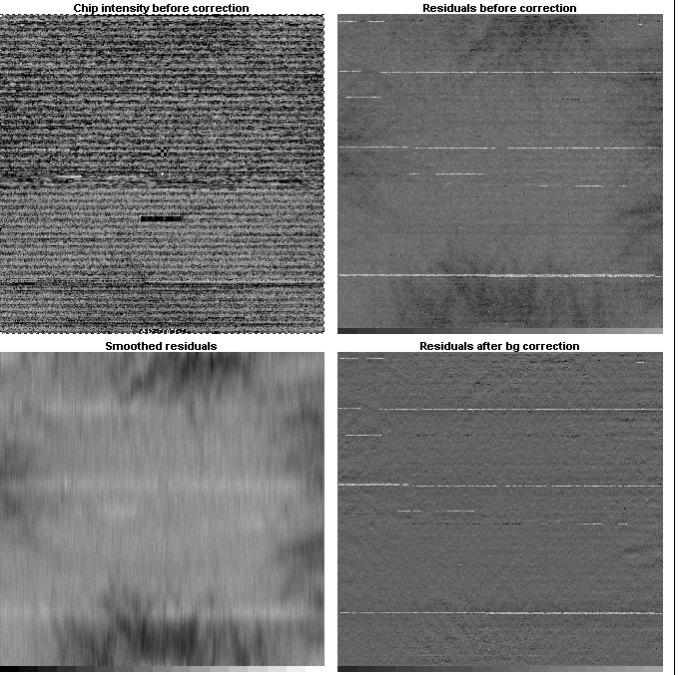
**Background Correction and Spatial normalization**. Background correction steps: chip image prior to background correction [top left], residual map prior to correction [top right], smoothed residuals map [bottom left], residual map after background correction [bottom right].

**Figure 4:**
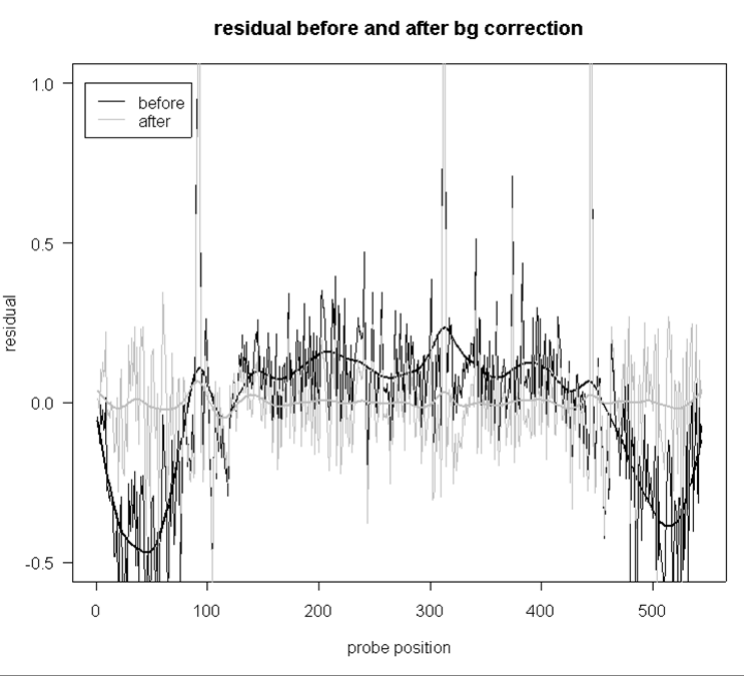
**Background Correction and Spatial normalization**. Effect of background correction on residuals. Black solid line shows the smoothed residuals before correction, gray lines represents the residuals after correction.

Our preliminary analysis revealed strong effects of spatial non-uniformity of chip intensity on copy number prediction. It appears that for these experiments, correction for spatial artifacts is the critical step in the analysis. Due to the nature of the experiment, it can be assumed that the target concentration remains the same unless a region of amplification or deletion occurs in a given sample. Hence, for a given position, a deviation from the median log-intensity should be randomly distributed around zero. The observed spatial artifacts were usually contiguous and stretched over significant areas covering multiple probes. This systematic local deviation from the median profile could be detected and then removed. We addressed this problem by constructing a median profile of the chips using normalized data for the entire dataset. Then we generated two-dimensional residual maps for each chip and applied spatial smoothing using cubic splines; see Figures 3 and 4.

### Signal transformation

**Figure 5:**
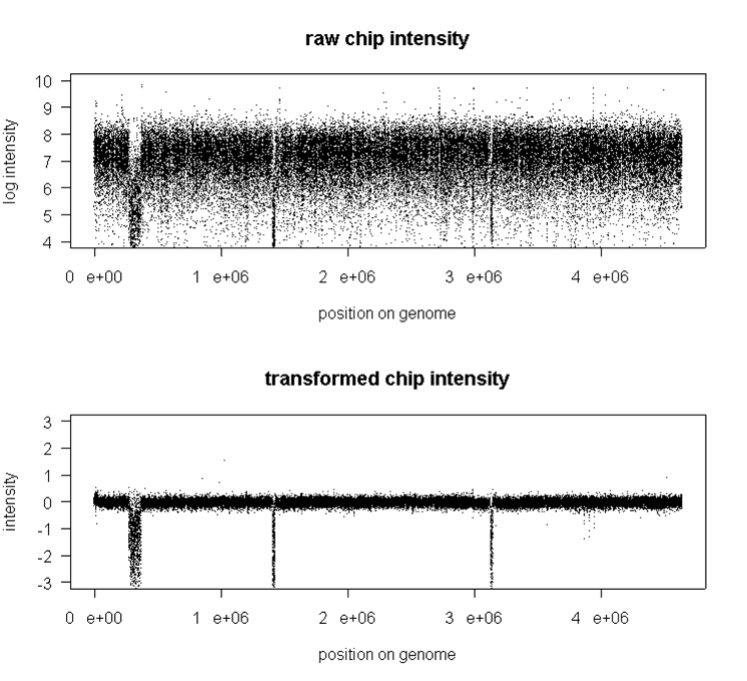
**Signal Transformation**. Result of subtracting probe effect from raw signal. Raw signal [upper panel] and transformed signal [lower panel].

Analysis of raw background-corrected signal intensities revealed a strong sequence-dependent probe effect characteristic of all expression microarray data. Raw signal intensities yield a wide distribution, heavily skewed towards low values. A common approach to modeling the probe effect is to fit a log-additive model with normal error to the background-adjusted and normalized probe intensities. We used a similar model in fitting our probe intensity data; see Methods section. The result of this procedure is shown in Figure 5, which demonstrates a dramatic reduction in signal variability across all regions. Removing probe-specific variation enhances sensitivity and allows for superior detection of genomic alterations. It was noticed that deleted regions appear to be noisier, due to a less efficient prediction of non-specific hybridization that is a function of many factors such as random cross-hybridizations that are difficult to predict.

**Figure 6:**
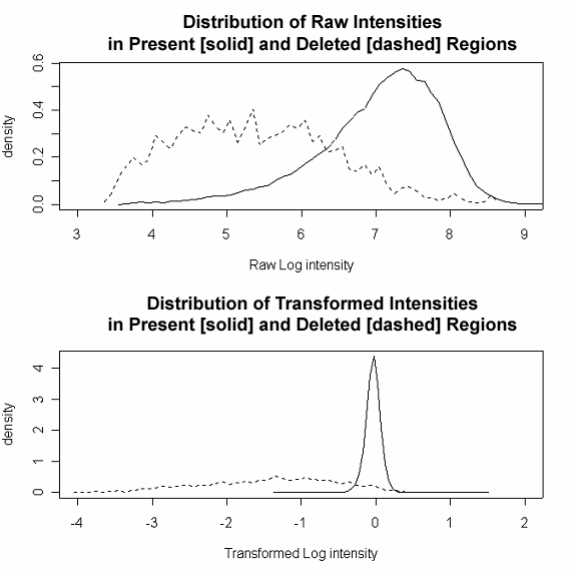
**Signal Transformation**. Distribution of log raw [top panel] and log transformed [bottom panel] intensity for probes in present (solid) and deleted (dashed) genomic regions.

Figure 6 shows the distribution of signal for the probes in present and deleted regions for raw and transformed data. We observe that the distributions in the top panel (raw data) for deleted and present regions share a significant overlap and thus present a challenging problem when identifying these regions. The lower panel, however, shows that transformation leads to a narrowing of the distribution of the signal that is densely located around 0. Also, the distribution of the transformed data shows significantly reduced overlap compared to the raw data. This observation is important for the HMM model fit described later.

### HMM-based DNA copy number inference

The goals in our experiment include accurate detection of chromosomal changes (while controlling false positive calls) and precise inference of the boundaries of copy number alterations (identifiying the breakpoints) in each GASP mutant. Although the major aberrations in a genome are frequently evident by inspection, several approaches have been developed to improve detection in the face of measurement noise. The simplest apply thresholds. If the ratio profile has only a few well-spaced ratio levels, then thresholds can be chosen by examination of the distribution of all measured ratios [21]. Use of smoothing by averaging the ratios on neighboring array elements improves the behavior of thresholding but blurs the locations of boundaries and reduces the amplitude of aberrations involving fewer elements than the smoothing window. More sophisticated analytical approaches rely on the fact that copy-number changes involve chromosome segments, and so copy numbers at contiguous loci should be identical, except for an occasional abrupt step to a new plateau. These methods assess statistically the status of each array element in the context of its neighbors [22-26].

Despite a general similarity, this study is different to classical arrayCGH studies in cancer research, where impurities in the sample and intrinsic heterogeneity of copy number among cells present a greater challenge to algorithms for inferring copy numbers and breakpoints. Even if the underlying biological process is discrete (counting relative copy numbers of DNA sequences), the signal in a classical arrayCGH analysis is viewed as being continuous, since possible values for chromosomal copy number in the test sample may vary considerably. This is especially true in the case of clinical tumor samples that contain mixtures of different cell types. In contrast, each DNA sample in this study was harvested from a single homogeneous *E. coli *colony, yielding changes in signal reflecting discrete changes in DNA copy number. We chose to use a Hidden Markov Model (HMM) [27,28] to distinguish genuine copy number changes from random microarray noise and to localize the start and end points of each copy number alteration. A key feature is the ability of the HMM to make correct inferences in regions where the data show high variance and might otherwise lead to mistaken conclusions [6,27]. A limitation of the HMM-based methods in the usual arrayCGH setting is that they assume invariance of the true hybridization signal intensity along chromosomal regions with the same copy number; however, the experimental design of our study makes this assumption reasonable. Another argument for choosing an HMM is its relative ease of interpretation. A model was fitted to a pre-assigned state space, i.e. deletion/no change/amplification, while for methods that do not control the number of possible states a significant post-processing effort is required.

**Figure 7:**
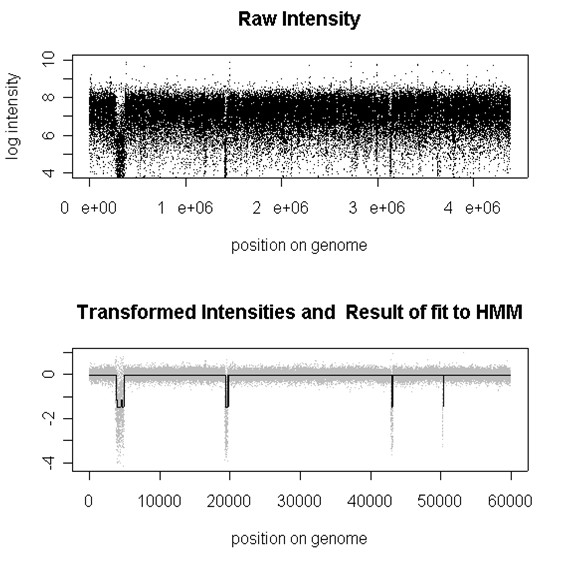
**HMM-based DNA Copy Number Inference**. A summary of signal processing steps using suggested set of routines. Upper panel shows signal before transformation (raw data), lower panel shows signal after transformation (grey dots) and result of fit to the HMM (black solid line).

Comparisons of several methods for segmenting conventional aCGH data are given in [29,30]. We visualize genomic alteration data according to their relative chromosome positions, so that the relationship between copy-number changes and their physical locations can be investigated in greater detail; see Figure 7.

## Conclusion

We have developed a method for inferring DNA copy number from experiments performed on Affymetrix high density expression chips. Our methodology includes a novel normalization method, specifically optimized for this type of data, that dramatically improved the quality of copy number extraction. With some modification our approach could be applied to gene expression profiling experiments and should significantly reduce noise. The suggested data transformation allows for efficient separation of the probe effect from different copy number signals, and the HMM-based method leads to accurate copy number detection.

The molecular mechanisms that generate large-scale deletions, duplications, and amplifications in evolving bacterial populations are not well understood. For example, at present we cannot determine whether homologous or non-homologous recombination events are responsible for creating deletion mutations. Since oligonucleotide-based arrays allow more precise mapping of the endpoints of these events, as more and more endpoints are mapped we may be able to determine the mechanisms leading to deletion events. Similar approaches may yield clues to the mechanisms of duplication and amplification as well.

## Methods

### Normalization method

**Figure 8:**
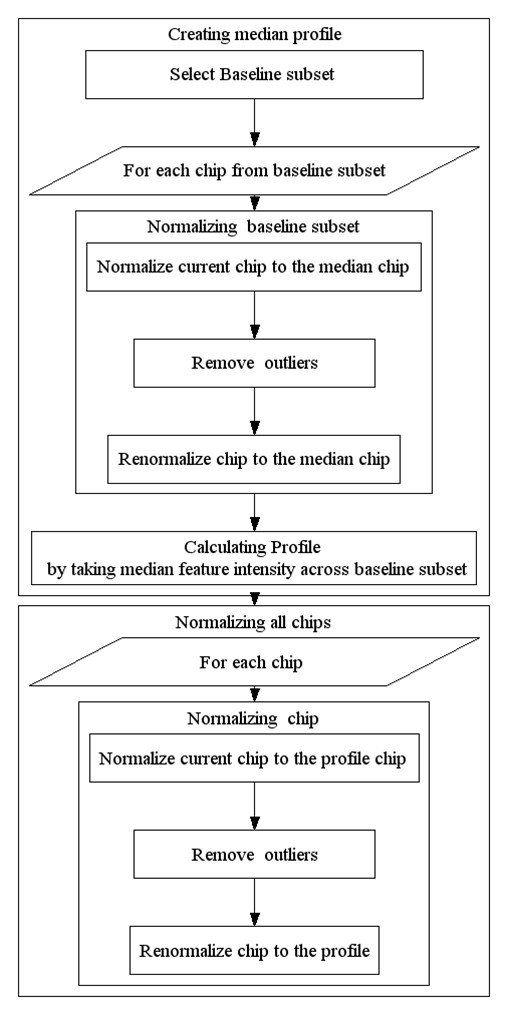
**Normalization Method**. Schematic description of normalization routine.

We introduce a novel multi-chip normalization method for Affymetrix-based aCGH data. This method has some similarity with the normalization techniques used in expression array analysis, however no assumptions of common distribution or existence of an invariant set are used. The key idea of the proposed routine is to derive a median profile using a set of specially selected chips, and then normalize all the chips to this profile using a smooth non-linear normalization function (such as loess). We make the assumption that the majority of DNA fragments do not change copy number in a given sample. Hence we can select fragments of chromosome that do not show deletions or amplifications and use them as an "invariant set" for subsequent non-linear robust normalization. Due to significant variation in median chip intensity, we select a set of 20 chips with median intensities close to the median chip intensity across the dataset as a "baseline set," used to extract the median intensity profile. We then proceed in an iterative manner: first, the entire set of feature intensities is used to define a normalization curve, then 10% of features with the largest residuals are removed and the reduced invariant set is used to refine the normalization curve. Then the baseline set is normalized to the median chip within the set and a median baseline profile is constructed by selecting the median intensity for each feature within the baseline set. Now that a set of features defining a normalization curve is selected and the baseline profile feature intensities are known, loess regression can be used to relate the baseline profile to each array to be normalized. Once loess normalization curves are generated, they are used to map probe intensities from the array to be normalized to the baseline. See Figure 8 for a schematic representation of the proposed normalization routine.

### Spatial normalization method

The Affymetrix GeneChip E. coli Antisense Genome Array is designed in such way that probes belonging to the same gene appear in consecutive order on the array. Furthermore, genes that are consecutive on a chromosome appear in the same order on the chip and features belonging to the neighboring genes are tiled immediately next to each other. Hence we observe long streaks of bright or dim runs in the amplified or deleted regions. This poses a serious problem since copy number and local background are intrinsically confounded by chip design, i.e. a smoother would recognize amplified or deleted regions as deviations from the median profile and treat them as local artifacts, removing them as a result of background correction. Since background correction is done prior to deletion/duplication detection, a smoother should be insensitive to copy number variation but be capable of effectively removing the background. In Affymetrix *E. coli *chips, probes are tiled along the x-axis of the array, resulting in the local background being confounded with copy number variation in the horizontal direction while being practically independent of copy number variation in the vertical direction. We observed that if the surface is analyzed vertically, local amplifications or deletions introduce minor effects on the local brightness. Two dimensional smoothers perform slightly worse at the boundaries of amplified regions (figure not shown). Hence we propose a vertical one-dimensional smoothing where the smoothing is done on a column-by-column basis. To obtain a spatial trend estimate, we use a cubic smoothing spline routine smooth.spline available in R [31]. Such vertical smoothing is likely to preserve amplified or deleted regions. The effect of this background correction are shown in Figure 4.

### Signal transformation method

The goal of the signal transformation described below is to eliminate the effect of hybridization properties of individual probes, so that the transformed signal intensity reflects true changes in copy number. For this purpose we assume that the normalized probe signal is the sum of two independent components, namely, specific and nonspecific:

*I *= *SP *+ *NS*

According to Affymetrix spike-in studies [32], the microarray hybridization signal is proportional to DNA concentration for a wide range of target concentrations, and thus we assume that the signal depends linearly on the target's concentration. The sample preparation protocol ensures a high concentration of specific target, so we can assume that non-specific binding is minimal compared to specific binding. Hence, in the presence of genomic DNA, probes mainly bind to specific DNA targets and the fraction of non-specific binding is insignificant. Provided that the chip is normalized and background-corrected, the statistical model of signal response to concentration for regions that are not deleted is:

Ii=εi⋅αiSP⋅c⋅si,
 MathType@MTEF@5@5@+=feaafiart1ev1aaatCvAUfKttLearuWrP9MDH5MBPbIqV92AaeXatLxBI9gBaebbnrfifHhDYfgasaacH8akY=wiFfYdH8Gipec8Eeeu0xXdbba9frFj0=OqFfea0dXdd9vqai=hGuQ8kuc9pgc9s8qqaq=dirpe0xb9q8qiLsFr0=vr0=vr0dc8meaabaqaciaacaGaaeqabaqabeGadaaakeaacqWGjbqsdaWgaaWcbaGaemyAaKgabeaakiabg2da9GGaciab=v7aLnaaBaaaleaacqWGPbqAaeqaaOGaeyyXICTae8xSde2aa0baaSqaaiabdMgaPbqaaiabdofatjabdcfaqbaakiabgwSixlabdogaJjabgwSixlabdohaZnaaBaaaleaacqWGPbqAaeqaaOGaeiilaWcaaa@452E@

where *I*_*i *_is the background-corrected and normalized probe intensity signal, *c *is concentration, *i *is probe index, αiSP
 MathType@MTEF@5@5@+=feaafiart1ev1aaatCvAUfKttLearuWrP9MDH5MBPbIqV92AaeXatLxBI9gBaebbnrfifHhDYfgasaacH8akY=wiFfYdH8Gipec8Eeeu0xXdbba9frFj0=OqFfea0dXdd9vqai=hGuQ8kuc9pgc9s8qqaq=dirpe0xb9q8qiLsFr0=vr0=vr0dc8meaabaqaciaacaGaaeqabaqabeGadaaakeaaiiGacqWFXoqydaqhaaWcbaGaemyAaKgabaGaem4uamLaemiuaafaaaaa@3232@ is the probe affinity to the specific signal, *s*_*i *_is the copy number of a particular probe *i *and *ε *is an error term. Since *s*_*i *_≥ 1, taking logarithms gives

log⁡Ii=log⁡εi+log⁡αiSP+log⁡c+log⁡si.
 MathType@MTEF@5@5@+=feaafiart1ev1aaatCvAUfKttLearuWrP9MDH5MBPbIqV92AaeXatLxBI9gBaebbnrfifHhDYfgasaacH8akY=wiFfYdH8Gipec8Eeeu0xXdbba9frFj0=OqFfea0dXdd9vqai=hGuQ8kuc9pgc9s8qqaq=dirpe0xb9q8qiLsFr0=vr0=vr0dc8meaabaqaciaacaGaaeqabaqabeGadaaakeaacyGGSbaBcqGGVbWBcqGGNbWzcqWGjbqsdaWgaaWcbaGaemyAaKgabeaakiabg2da9iGbcYgaSjabc+gaVjabcEgaNHGaciab=v7aLnaaBaaaleaacqWGPbqAaeqaaOGaey4kaSIagiiBaWMaei4Ba8Maei4zaCMae8xSde2aa0baaSqaaiabdMgaPbqaaiabdofatjabdcfaqbaakiabgUcaRiGbcYgaSjabc+gaVjabcEgaNjabdogaJjabgUcaRiGbcYgaSjabc+gaVjabcEgaNjabdohaZnaaBaaaleaacqWGPbqAaeqaaOGaeiOla4caaa@5590@

We define a factor that represents individual probe hybridization properties as

vi=E(log⁡Ii|si=1)=log⁡αiSP+log⁡c.
 MathType@MTEF@5@5@+=feaafiart1ev1aaatCvAUfKttLearuWrP9MDH5MBPbIqV92AaeXatLxBI9gBaebbnrfifHhDYfgasaacH8akY=wiFfYdH8Gipec8Eeeu0xXdbba9frFj0=OqFfea0dXdd9vqai=hGuQ8kuc9pgc9s8qqaq=dirpe0xb9q8qiLsFr0=vr0=vr0dc8meaabaqaciaacaGaaeqabaqabeGadaaakeaacqWG2bGDdaWgaaWcbaGaemyAaKgabeaakiabg2da9iabdweafjabcIcaOiGbcYgaSjabc+gaVjabcEgaNjabdMeajnaaBaaaleaacqWGPbqAaeqaaOGaeiiFaWNaem4Cam3aaSbaaSqaaiabdMgaPbqabaGccqGH9aqpcqaIXaqmcqGGPaqkcqGH9aqpcyGGSbaBcqGGVbWBcqGGNbWziiGacqWFXoqydaqhaaWcbaGaemyAaKgabaGaem4uamLaemiuaafaaOGaey4kaSIagiiBaWMaei4Ba8Maei4zaCMaem4yamMaeiOla4caaa@52A4@

For *r *= 1, 2,..., if *s*_*i *_= *r *we assume

log⁡Ii−vi~N(log⁡r,σr2)
 MathType@MTEF@5@5@+=feaafiart1ev1aaatCvAUfKttLearuWrP9MDH5MBPbIqV92AaeXatLxBI9gBaebbnrfifHhDYfgasaacH8akY=wiFfYdH8Gipec8Eeeu0xXdbba9frFj0=OqFfea0dXdd9vqai=hGuQ8kuc9pgc9s8qqaq=dirpe0xb9q8qiLsFr0=vr0=vr0dc8meaabaqaciaacaGaaeqabaqabeGadaaakeaacyGGSbaBcqGGVbWBcqGGNbWzcqWGjbqsdaWgaaWcbaGaemyAaKgabeaakiabgkHiTiabdAha2naaBaaaleaacqWGPbqAaeqaaOGaeiOFa4NaemOta4KaeiikaGIagiiBaWMaei4Ba8Maei4zaCMaemOCaiNaeiilaWccciGae83Wdm3aa0baaSqaaiabdkhaYbqaaiabikdaYaaakiabcMcaPaaa@468F@

These equations are true for regions that are not deleted. If a DNA region undergoes deletion, target DNA for that region is missing and thus specific binding is absent for the probes spanning that region. Hence the signal for deleted regions is entirely due to non-specific random binding, and the fraction of specific binding is zero. A model for a deleted region (*s*_*i *_= 0) is then:

log⁡Ii=log⁡εi+log⁡αiNS+log⁡c,
 MathType@MTEF@5@5@+=feaafiart1ev1aaatCvAUfKttLearuWrP9MDH5MBPbIqV92AaeXatLxBI9gBaebbnrfifHhDYfgasaacH8akY=wiFfYdH8Gipec8Eeeu0xXdbba9frFj0=OqFfea0dXdd9vqai=hGuQ8kuc9pgc9s8qqaq=dirpe0xb9q8qiLsFr0=vr0=vr0dc8meaabaqaciaacaGaaeqabaqabeGadaaakeaacyGGSbaBcqGGVbWBcqGGNbWzcqWGjbqsdaWgaaWcbaGaemyAaKgabeaakiabg2da9iGbcYgaSjabc+gaVjabcEgaNHGaciab=v7aLnaaBaaaleaacqWGPbqAaeqaaOGaey4kaSIagiiBaWMaei4Ba8Maei4zaCMae8xSde2aa0baaSqaaiabdMgaPbqaaiabd6eaojabdofatbaakiabgUcaRiGbcYgaSjabc+gaVjabcEgaNjabdogaJjabcYcaSaaa@4D88@

and we assume

log⁡Ii−vi~N(log⁡αiNS−log⁡αiSP,σ02),
 MathType@MTEF@5@5@+=feaafiart1ev1aaatCvAUfKttLearuWrP9MDH5MBPbIqV92AaeXatLxBI9gBaebbnrfifHhDYfgasaacH8akY=wiFfYdH8Gipec8Eeeu0xXdbba9frFj0=OqFfea0dXdd9vqai=hGuQ8kuc9pgc9s8qqaq=dirpe0xb9q8qiLsFr0=vr0=vr0dc8meaabaqaciaacaGaaeqabaqabeGadaaakeaacyGGSbaBcqGGVbWBcqGGNbWzcqWGjbqsdaWgaaWcbaGaemyAaKgabeaakiabgkHiTiabdAha2naaBaaaleaacqWGPbqAaeqaaOGaeiOFa4NaemOta4KaeiikaGIagiiBaWMaei4Ba8Maei4zaCgcciGae8xSde2aa0baaSqaaiabdMgaPbqaaiabd6eaojabdofatbaakiabgkHiTiGbcYgaSjabc+gaVjabcEgaNjab=f7aHnaaDaaaleaacqWGPbqAaeaacqWGtbWucqWGqbauaaGccqGGSaalcqWFdpWCdaqhaaWcbaGaeGimaadabaGaeGOmaidaaOGaeiykaKIaeiilaWcaaa@5592@

where αiNS
 MathType@MTEF@5@5@+=feaafiart1ev1aaatCvAUfKttLearuWrP9MDH5MBPbIqV92AaeXatLxBI9gBaebbnrfifHhDYfgasaacH8akY=wiFfYdH8Gipec8Eeeu0xXdbba9frFj0=OqFfea0dXdd9vqai=hGuQ8kuc9pgc9s8qqaq=dirpe0xb9q8qiLsFr0=vr0=vr0dc8meaabaqaciaacaGaaeqabaqabeGadaaakeaaiiGacqWFXoqydaqhaaWcbaGaemyAaKgabaGaemOta4Kaem4uamfaaaaa@322E@ is a probe affinity to the non-specific signal.

Thus, for all regions that are not deleted, the transformed signal *T*_*i *_= log(*I*_*i*_) - *v*_*i *_is independent of individual probe characteristics and is related to the copy number by (1) and (2). Note that for deleted regions the signal is shifted toward negative values since log⁡αiNS<log⁡αiSP
 MathType@MTEF@5@5@+=feaafiart1ev1aaatCvAUfKttLearuWrP9MDH5MBPbIqV92AaeXatLxBI9gBaebbnrfifHhDYfgasaacH8akY=wiFfYdH8Gipec8Eeeu0xXdbba9frFj0=OqFfea0dXdd9vqai=hGuQ8kuc9pgc9s8qqaq=dirpe0xb9q8qiLsFr0=vr0=vr0dc8meaabaqaciaacaGaaeqabaqabeGadaaakeaacyGGSbaBcqGGVbWBcqGGNbWziiGacqWFXoqydaqhaaWcbaGaemyAaKgabaGaemOta4Kaem4uamfaaOGaeyipaWJagiiBaWMaei4Ba8Maei4zaCMae8xSde2aa0baaSqaaiabdMgaPbqaaiabdofatjabdcfaqbaaaaa@40F2@. In practice *v*_*i *_can be estimated as a median feature intensity across the dataset. Here, we assume that for a given position on the genome, the median intensity across the dataset corresponds to a single DNA copy number, e.g. no genomic alterations occur in more that 50% of the samples for a given position. Hence, signal transformation can be performed by subtracting the median profile from the chip of interest on the natural log scale, thus subtracting *v*_*i *_from *I*_*i*_. The transformed data show greatly improved consistency in probe intensity patterns and significant decrease in probe-sequence-specific variation; see Results section.

### Inferring DNA copy number

We fit an HMM to the vector of normalized, background-corrected and transformed probe intensities for each GASP mutant. For each chip we determine the number of states and define the boundaries of the derived states. The relevant theory as well as a detailed description of the HMM routine used is given in [33].

We can characterize the genomic profiles using two types of genomic change (amplification or deletion) and a 'no genomic alterations' state. In our HMM we assumed that some regions are amplified with a different amplification factor, hence we used a model with up to five states. Further increase in the number of states does not seem to be necessary; computational cost is proportional to the square of the number of states, and we have not observed more than four states in the sample of 116 different morphotypes.

Non-uniform probe spacings across the genome pose a significant problem for designing a proper model. Affymetrix *E. coli *chips have two types of probesets, corresponding to gene coding regions and intergenic (IG) regions. Those probesets have a significant difference in design. In particular, probes for gene-coding probesets are sliced from genomic sequence in a non-overlapping manner and probes are usually spaced by 25 bp. In contrast, IG probes are selected from the sequence with a shift of one nucleotide and thus overlap, so that the whole IG probeset covers a region of about 40 bp. Hence consecutive IG probes cannot be treated as independent measurements due to this significant overlap. Instead, IG probes within each probeset might better be considered as replicated measurements of the same signal. Incorporating this design feature would significantly complicate the analysis. Given that IG probesets constitute only 5% of coding probesets coverage, we did not implement this approach. Hence we exclude observations from IG probesets from our model.

As mentioned earlier, probes within a given coding gene probeset are spaced evenly, however the distance between probesets is significantly larger (about 1 kb). We hypothesize that the probability of observing a breakpoint within an interval is uniform and proportional to the length of the interval. Our preliminary analysis, omitted here, supported this claim and showed that elements of a spacing-dependent transition matrix converge rapidly to some constant values within about a hundred bases and that gap-length dependence can be assumed constant for gaps of > 300 bp with a high degree of accuracy. Thus instead of implementing a computationally intensive non-homogenous model with transition matrix a function of the distance between neighboring probes [28], we applied a practical approximation where one of two possible transition matrices is chosen based on the distance to the next observation. To incorporate this design feature, we used two constant transition matrices – one representing transition probabilities between probes within a probeset and the other transition probabilities corresponding to transitions between probesets. The probability of jumping from state to state is small enough to ensure that the expected number of transitions is of order one.

We observed that the signal for no-genomic-alteration and amplification states have symmetrical distributions with heavy tails, while the signal for deletion states has a more skewed shape. This effect is not accounted for in formula (1). Additionally, due to imperfect normalization, transformed intensities for some of the chips exhibited distribution shift away from the zero mean assumed in the model. Hence, in order to account for these effects, we avoided using predefined model parameters and instead resorted to fitting them iteratively. To model this we used a mixture of two Gaussian distributions, where means and variances were obtained for each chip individually during the training step. Initial values were provided according to formula (1). These values were then iteratively re-estimated by the Baum-Welch procedure. We use these parameter estimates for the final segmentation step, which is performed using the Viterbi algorithm. R code that implements the HMM is provided in the supplementary material section.

## Authors' contributions

DS carried out an implementation of the normalization and spatial correction. DS and DA performed the HMM computations. DA drafted the manuscript. ST helped to develop the HMM model, edited the paper and supervised the analysis. MS and SEF generated the microarray data. All authors read and approved the final manuscript.

## References

[B1] Kallioniemi OP, Kallioniemi A, Sudar D, Rutovitz D, Gray JW, Waldman F, Pinkel D (1993). Comparative genomic hybridization: a rapid new method for detecting and mapping DNA amplification in tumors. Semin Cancer Biol.

[B2] Solinas-Toldo S, Lampel S, Stilgenbauer S, Nickolenko J, Benner A, Dohner H, Cremer T, Lichter P (1997). Matrix-based comparative genomic hybridization: biochips to screen for genomic imbalances. Genes Chromosomes Cancer.

[B3] Pollack JR, Perou CM, Alizadeh AA, Eisen MB, Pergamenschikov A, Williams CF, Jeffrey SS, Botstein D, Brown PO (1999). Genome-wide analysis of DNA copy-number changes using cDNA microarrays. Nat Genet.

[B4] Heiskanen MA, Bittner ML, Chen Y, Khan J, Adler KE, Trent JM, Meltzer PS (2000). Detection of gene amplification by genomic hybridization to cDNA microarrays. Cancer Res.

[B5] Huang J, Wei W, Zhang J, Liu G, Bignell GR, Stratton MR, Futreal PA, Wooster R, Jones KW, Shapero MH (2004). Whole genome DNA copy number changes identified by high density oligonucleotide arrays. Hum Genomics.

[B6] Janne PA, Li C, Zhao X, Girard L, Chen TH, Minna J, Christiani DC, Johnson BE, Meyerson M (2004). High-resolution single-nucleotide polymorphism array and clustering analysis of loss of heterozygosity in human lung cancer cell lines. Oncogene.

[B7] Lucito R, Healy J, Alexander J, Reiner A, Esposito D, Chi M, Rodgers L, Brady A, Sebat J, Troge J, West JA, Rostan S, Nguyen KC, Powers S, Ye KQ, Olshen A, Venkatraman E, Norton L, Wigler M (2003). Representational oligonucleotide microarray analysis: a high-resolution method to detect genome copy number variation. Genome Res.

[B8] Bignell GR, Huang J, Greshock J, Watt S, Butler A, West S, Grigorova M, Jones KW, Wei W, Stratton MR, Futreal PA, Weber B, Shapero MH, Wooster R (2004). High-resolution analysis of DNA copy number using oligonucleotide microarrays. Genome Res.

[B9] Carvalho B, Ouwerkerk E, Meijer GA, Ylstra B (2004). High resolution microarray comparative genomic hybridisation analysis using spotted oligonucleotides. J Clin Pathol.

[B10] Sebat J, Lakshmi B, Troge J, Alexander J, Young J, Lundin P, Maner S, Massa H, Walker M, Chi M, Navin N, Lucito R, Healy J, Hicks J, Ye K, Reiner A, Gilliam TC, Trask B, Patterson N, Zetterberg A, Wigler M (2004). Large-scale copy number polymorphism in the human genome. Science.

[B11] Redon R, Ishikawa S, Fitch KR, Feuk L, Perry GH, Andrews TD, Fiegler H, Shapero MH, Carson AR, Chen W, Cho EK, Dallaire S, Freeman JL, Gonzalez JR, Gratacos M, Huang J, Kalaitzopoulos D, Komura D, MacDonald JR, Marshall CR, Mei R, Montgomery L, Nishimura K, Okamura K, Shen F, Somerville MJ, Tchinda J, Valsesia A, Woodwark C, Yang F, Zhang J, Zerjal T, Zhang J, Armengol L, Conrad DF, Estivill X, Tyler-Smith C, Carter NP, Aburatani H, Lee C, Jones KW, Scherer SW, Hurles ME (2006). Global variation in copy number in the human genome. Nature.

[B12] Porwollik S, Wong RM, Helm RA, Edwards KK, Calcutt M, Eisenstark A, McClelland M (2004). DNA amplification and rearrangements in archival Salmonella enterica serovar Typhimurium LT2 cultures. J Bacteriol.

[B13] Whoriskey SK, Nghiem VH, Leong PM, Masson JM, Miller JH (1987). Genetic rearrangements and gene amplification in *Escherichia coli*: DNA sequences at the junctures of amplified gene fusions. Genes Dev.

[B14] Anderson RP, Roth JR (1977). Tandem genetic duplications in phage and bacteria. Annu Rev Microbiol.

[B15] Finkel SE (2006). Long-term survival during stationary phase: evolution and the GASP phenotype. Nat Rev Microbiol.

[B16] Zambrano MM, Siegele DA, Almiron M, Tormo A, Kolter R (1993). Microbial competition: *Escherichia coli* mutants that take over stationary phase cultures. Science.

[B17] Zinser ER, Kolter R (2004). *Escherichia coli* evolution during stationary phase. Res Microbiol.

[B18] Zinser ER, Schneider D, Blot M, Kolter R (2003). Bacterial evolution through the selective loss of beneficial genes. Trade-offs in expression involving two loci. Genetics.

[B19] Finkel SE, Kolter R (1999). Evolution of microbial diversity during prolonged starvation. Proc Natl Acad Sci USA.

[B20] Salamon H, Kato-Maeda M, Small PM, Drenkow J, Gingeras TR (2000). Detection of deleted genomic DNA using a semiautomated computational analysis of GeneChip data. Genome Res.

[B21] Hodgson G, Hager JH, Volik S, Hariono S, Wernick M, Moore D, Nowak N, Albertson DG, Pinkel D, Collins C, Hanahan D, Gray JW (2001). Genome scanning with array CGH delineates regional alterations in mouse islet carcinomas. Nat Genet.

[B22] Olshen AB, Venkatraman ES, Lucito R, Wigler M (2004). Circular binary segmentation for the analysis of array-based DNA copy number data. Biostatistics.

[B23] Hupé P, Stransky N, Thiery JP, Radvanyi F, Barillot E (2004). Analysis of array CGH data: from signal ratio to gain and loss of DNA regions. Bioinformatics.

[B24] Jong K, Marchiori E, Meijer G, van der Vaart A, Ylstra B (2004). Breakpoint identification and smoothing of array comparative genomic hybridisation data. Bioinformatics.

[B25] Wang P, Kim Y, Pollack J, Narasimhan B, Tibshirani R (2005). A method for calling gains and losses in array CGH data. Biostatistics.

[B26] Hsu L, Self SG, Grove D, Randolph T, Wang K, Delrow JJ, Loo L, Porter P (2005). Denoising array-based comparative genomic hybridization data using wavelets. Biostatistics.

[B27] Fridlyand J, Snijders A, Pinkel D, Albertson D, Jain A (2004). Hidden Markov models approach to the analysis of array CGH data. Journal of Multivariate Analysis.

[B28] Marioni JC, Thorne NP, Tavaré S (2006). BioHMM: a heterogeneous hidden Markov model for segmenting array CGH data. Bioinformatics.

[B29] Willenbrock H, Fridlyand J (2005). A comparison study: applying segmentation to array CGH data for downstream analyses. Bioinformatics.

[B30] Lai WR, Johnson MD, Kucherlapati R, Park PJ (2005). Comparative analysis of algorithms for identifying amplifications and deletions in array CGH data. Bioinformatics.

[B31] R Development Core Team (2005). R: A language and environment for statistical computing.

[B32] Affymetrix (2001). Latin Square Data for Expression Algorithm Assessment. http://www.affymetrix.com/support/technical/sample data/datasets.affx.

[B33] Rabiner LR (1989). A tutorial on hidden Markov models and selected applications in speech recognition. Proceedings of the IEEE.

